# Synergistic effect of IL-12 and IL-18 induces TIM3 regulation of γδ T cell function and decreases the risk of clinical malaria in children living in Papua New Guinea

**DOI:** 10.1186/s12916-017-0883-8

**Published:** 2017-06-15

**Authors:** Louis Schofield, Lisa J. Ioannidis, Stephan Karl, Leanne J. Robinson, Qiao Y. Tan, Daniel P. Poole, Inoni Betuela, Danika L. Hill, Peter M. Siba, Diana S. Hansen, Ivo Mueller, Emily M. Eriksson

**Affiliations:** 1 Walter and Eliza Hall Institute of Medical Research, Division of Population Health and Immunity, Melbourne, VIC 3052 Australia; 2 Australian Institute of Tropical Health and Medicine, James Cook University, Townsville, QLD 4811 Australia; 3 Department of Medical Biology, The University of Melbourne, Melbourne, VIC 3052 Australia; 4 Papua New Guinea Institute of Medical Research, Goroka and Madang, Papua New Guinea; 5 Burnet Institute, Melbourne, VIC 3004 Australia; 6 Monash Institute of Pharmaceutical Sciences, Monash University, Melbourne, VIC 3052 Australia; 7 Department of Anatomy and Neuroscience, The University of Melbourne, Melbourne, VIC 3010 Australia; 8 School of Veterinary and Biomedical Sciences, James Cook University, Townsville, QLD 4811 Australia; 9 The Walter and Eliza Hall Institute of Medical Research, 1G Royal Parade, Melbourne, VIC 3052 Australia

**Keywords:** γδ T cells, TIM3, IL-12, IL-18, Malaria, *Plasmodium*

## Abstract

**Background:**

γδ T cells are important for both protective immunity and immunopathogenesis during malaria infection. However, the immunological processes determining beneficial or detrimental effects on disease outcome remain elusive. The aim of this study was to examine expression and regulatory effect of the inhibitory receptor T-cell immunoglobulin domain and mucin domain 3 (TIM3) on γδ T cells. While TIM3 expression and function on conventional αβ T cells have been clearly defined, the equivalent characterization on γδ T cells and associations with disease outcomes is limited. This study investigated the functional capacity of TIM3+ γδ T cells and the underlying mechanisms contributing to TIM3 upregulation and established an association with malaria disease outcomes.

**Methods:**

We analyzed TIM3 expression on γδ T cells in 132 children aged 5–10 years living in malaria endemic areas of Papua New Guinea. TIM3 upregulation and effector functions of TIM3+ γδ T cells were assessed following in vitro stimulation with parasite-infected erythrocytes, phosphoantigen and/or cytokines. Associations between the proportion of TIM3-expressing cells and the molecular force of infection were tested using negative binomial regression and in a Cox proportional hazards model for time to first clinical episode. Multivariable analyses to determine the association of TIM3 and IL-18 levels were conducted using general linear models. Malaria infection mouse models were utilized to experimentally investigate the relationship between repeated exposure and TIM3 upregulation.

**Results:**

This study demonstrates that even in the absence of an active malaria infection, children of malaria endemic areas have an atypical population of TIM3-expressing γδ T cells (mean frequency TIM3+ of total γδ T cells 15.2% ± 12). Crucial factors required for γδ T cell TIM3 upregulation include IL-12/IL-18, and plasma IL-18 was associated with TIM3 expression (*P* = 0.002). Additionally, we show a relationship between TIM3 expression and infection with distinct parasite clones during repeated exposure. TIM3+ γδ T cells were functionally impaired and were associated with asymptomatic malaria infection (hazard ratio 0.54, *P* = 0.032).

**Conclusions:**

Collectively our data demonstrate a novel role for IL-12/IL-18 in shaping the innate immune response and provide fundamental insight into aspects of γδ T cell immunoregulation. Furthermore, we show that TIM3 represents an important γδ T cell regulatory component involved in minimizing malaria symptoms.

**Electronic supplementary material:**

The online version of this article (doi:10.1186/s12916-017-0883-8) contains supplementary material, which is available to authorized users.

## Background

Immunoregulation is an essential part of the immune response and ensures that a comprehensive and protective response is elicited, but with limited damage to the host. Immunoregulation can be achieved by check-point proteins that either enhance or inhibit cell reactivity [1]. Inappropriate expression of these proteins can therefore have detrimental consequences for immune responses to infection and also affect pathogenesis. Thus, blocking of check-point proteins to restore functional capacity of effector cells has been explored as potential immunotherapy for chronic viral infections and cancers [2–8]. The expression of immunoregulatory proteins on conventional T cells has been recently shown in both malaria rodent infection models and in malaria-infected individuals, where inhibitory therapies of immunoregulatory proteins resulted in enhanced parasite clearance [9–13]. However, the effect of continuous malaria exposure on immunoregulation among the *innate* cell compartment remains a critically under-investigated aspect of malaria immunology.

γδ T cells are a subset of T cells that express a distinct T cell receptor (TCR). These cells are considered to be part of the innate/intermediate immune system due to their ability to respond rapidly to non-peptide antigens without the requirement of major histocompatibility complex (MHC) presentation. Substantial evidence indicates that γδ T cells mediate essential protection against a number of pathogens including *Plasmodium* [14–19] where *Plasmodium* stimulation of γδ T cells involves metabolites of the 1-deoxy-d-xylulose 5-phosphate (DOXP) pathway [20]. While immunity to malaria requires a multifaceted network of cell interactions and cytokine production involving both innate and adaptive immune responses, γδ T cells have been shown to contribute to key processes associated with beneficial outcomes [21, 22]. Mouse studies demonstrate that the frequency of γδ T cells is significantly increased during malaria infection and they provide protective immunity via interferon gamma (IFN-γ) production and control of parasitemia [21, 23, 24]. Similarly, γδ T cells are an important early source of IFN-γ in malaria-infected individuals, which is associated with reduced risk of clinical disease [25–30]. Furthermore, inhibition of intracellular parasite growth and granulysin-dependent cytotoxic activity against released blood stage merozoites have been demonstrated [14, 31–33]. However, in addition to a protective role during malaria, γδ T cells were also suggested to contribute to pathogenesis. This is supported by observations that depletion of γδ T cells protected mice from developing cerebral malaria in a *P. berghei* ANKA mouse model [34] and that γδ T cells were found to be one of the predominant sources of cytokines and chemokines associated with severe malaria in malaria-infected individuals [29]. Although numerous studies describe activation of γδ T cells in response to malaria, the understanding of how these cells are regulated is remarkably limited.

T-cell immunoglobulin domain and mucin domain 3 (TIM3) is a relatively recently described immunoregulatory protein that belongs to the TIM protein family. In humans this family consists of TIM1, TIM3, and TIM4, whereas mice have an additional protein, TIM2 [35–38]. TIM3 is expressed by T cells, dendritic cells (DCs), natural killer (NK) cells, and monocytes and interacts with galectin-9 [39]. TIM3 is generally referred to as a negative regulator, but TIM3 expression can affect different functions in the innate and the adaptive immune system and on different cells. In mice, engagement of TIM3 on conventional αβ T cells results in apoptosis and loss of effector T cells [39], whereas TIM3 expression on human T cells is associated with functional exhaustion [4, 39–41]. In contrast, TIM3 is thought to be a maturation marker on human NK cells [42] and is also abundantly expressed on monocytes regulating cytokine production in these cells [43, 44]. Recently TIM3 was observed to be upregulated in mice during acute *Plasmodium* infection [13, 45]. TIM3 was found to be expressed by conventional T cells and NK cells, and in vivo blocking of TIM3 resulted in enhanced parasite clearance [13]. Furthermore, TIM3-expressing CD4+ and CD8+ αβ T cells were observed in individuals during acute *P. vivax* infection but were undetectable following treatment [46].

In contrast, the effect of TIM3 expression on γδ T cells has only recently started to receive attention [47, 48]. In malaria, the biological relevance of TIM3 expression on γδ T cell function for clinical outcomes has not previously been investigated. Recent findings suggest that dysfunctional Vδ2 γδ T cells associated with malaria exposure induce tolerance to the *Plasmodium* parasite [47]. However, the precise immunological processes responsible for γδ T cell dysregulation remain elusive. Here, we specifically investigated associations between TIM3 and γδ T cell function during malaria as well as the factors that govern TIM3 upregulation. The roles TIM3 expression plays in the control of pathogenic mechanisms were also explored. Our main findings revealed that interleukin (IL)-12 in synergy with IL-18 are key factors required for TIM3 induction. Moreover, TIM3 expression renders cells functionally impaired, which is associated with reduced risk of clinical malaria. These findings provide novel insights into immune-specific processes involved in γδ T cell regulation and represent a major advancement in the field of γδ T cell biology.

## Methods

### Study site and subjects

Human blood samples from children aged 5–10 years were collected in a clinical trial (ClinicalTrials.gov registration: NCT02143934) conducted in 2009 and 2010 in five villages in East Sepik Province of Papua New Guinea (PNG), where both *P. falciparum* and *P. vivax* are endemic [49]. Children were randomized into two treatment groups of directly observed treatment (DOT) over a total of 27 days. The first group of children received chloroquine (CQ, days 1–3 of DOT), artemether-lumefantrine (Coartem®) (AL, days 11–13 of DOT), and primaquine (PQ, days 1–20 of DOT; 0.5 mg/kg per dose). The second group of children received CQ (days 1–3 of DOT), AL (days 11–13 of DOT), and a placebo (days 1–20 of DOT). The drug treatment was implemented to be able to quantify the contribution of *P. vivax* and *P. ovale* relapses to infection and disease during follow-up in an epidemiological study of the cohort [49]. The first treatment regime was designed to clear all parasites including *P. vivax* hypnozoite stages, whereas the second treatment regime cleared only blood stages. Venous bleeds and peripheral blood mononuclear cell (PBMC) isolation were performed following completion of drug treatment, and PBMCs were cryopreserved.

Children were actively followed for 8 months with finger-prick (250-μl) blood samples collected every 2 weeks for the first 12 weeks and every 4 weeks for the remainder of the follow-up period. In addition passive surveillance measures were implemented at local health centers, aid posts, and via the village health volunteer network. Febrile children were tested with a rapid diagnostic test (RDT), and a blood slide was collected. Symptomatic infections (those with fever and who tested positive by RDT and/or light microscopy) during follow-up were treated with AL. For RDT-negative children, the slides were read within 12 h. If the slides were positive, the children were treated the next day. If the slide was negative, the result was recorded but no further action taken. The collected blood samples were screened for infection with *Plasmodium* spp. by light microscopy and quantitative real-time PCR (qPCR). Slides were scored as light microscopy-positive for an individual *Plasmodium* species if the species was detected independently by at least two microscopists and/or if subsequent qPCR diagnosis confirmed the presence of the species. Slide discrepancies were adjudicated by a World Health Organization (WHO)-certified level 1 (expert) microscopist [49]. A generic qPCR was used to detect all *Plasmodium* species occurring in PNG*,* followed by subsequent species-specific qPCRs on *Plasmodium-*positive samples [50, 51].

A subset of PBMC samples from the children enrolled in the clinical trial (*n* = 132, of which *n* = 63 belonged to the primaquine drug-treated group and *n* = 69 belonged to the placebo group) were included in the current study. These children all had at least one *Plasmodium falciparum* infection verified by PCR during follow-up to ensure ongoing exposure. Of these, 50 individuals had a clinical episode during follow-up. A clinical episode of malaria was defined as febrile illness (axillary temperature of ≥37.5 °C, current or previous 48 h) plus the presence of *P. vivax* or *P. falciparum* parasites (any density) by light microscopy. PBMCs collected from 20 healthy blood donors by the Australian Red Cross were used as controls.

### Mouse infections

Female C57BL/6 mice aged 6–8 weeks were infected with 5 × 10^4^ *Plasmodium chabaudi*-infected red blood cells (iRBCs) intravenously or with 1 × 10^6^ *P. berghei* ANKA iRBCs intraperitoneally. *P. chabaudi*-infected mice were drug-treated on day 14 post-infection with CQ (6 μg/ml)- and pyrimethamine (70 μg/ml)-containing water for 5 days. Drug treatment on day 14 coincided with control of the infection and allowed for a whole infection cycle to be completed before drug treatment. *P. berghei*-infected mice were treated at day 5 post-infection to avoid progression to cerebral malaria. Drug treatment consisted of an intraperitoneal injection of CQ (10 mg/kg) and pyrimethamine (10 mg/kg) followed by CQ- and pyrimethamine-containing water for 5 days as described previously [52]. Livers and spleens were removed at different time points following completion of drug treatment. Untreated *P. chabaudi*-infected mice establish a submicroscopic chronic infection with intermittent detectable parasitemia peaks. Mice that were chronically infected with *P. chabaudi* were left untreated until day 98 after the initial infection. Mice infected multiple times (three consecutive infections (*P. chabaudi* only) or two consecutive infections (*P. chabaudi* and *P. berghei*)) were drug-treated and then re-infected on day 14 post-completion of drug treatment to allow the immune cells to return to steady state (Additional file 1: Figure S1).

### Parasite lines and cultures

Parasite lines 3D7 and XIE were cultured in human red blood cells, and trophozoite-stage parasites were isolated as described previously [29, 53]. XIE parasites were snap frozen in 15% glycerol in phosphate-buffered saline (PBS) and thawed by sequential addition of 12%, 1.8%, and 0.9% NaCl and subsequently used for stimulation of cohort samples.

### Flow cytometry

PBMCs (5 × 10^5^) were stained with antibody cocktails in FACS buffer (PBS containing 0.5% bovine serum albumin and 2 mM ethylenediaminetetraacetic acid) on ice for 30 min. The human antibodies used were fluorescein isothiocyanate (FITC)-conjugated anti-γδTCR (clone 11 F2, BD Biosciences, San Jose, CA, USA), Qdot 605-conjugated anti-CD27-, Qdot 655-conjugated Streptavidin (Invitrogen, Carlsbad, CA, USA), PE-Texas Red (ECD)-conjugated anti-CD3 (clone UCHT1, Beckman Coulter, Brea, CA, USA), Brilliant Violet 421-conjugated anti-CD16-(clone 3G8), Brilliant Violet 711-conjugated anti-CD45RA (clone HI100, both from Biolegend, San Diego, CA, USA), and phycoerythrin (PE)-conjugated anti-TIM3 (clone FAB2365P from R&D Systems Minneapolis, MN, USA). The mouse antibodies used were FITC-comjugated anti-CD3 (clone 145-2C11), PE-conjugated anti-TIM3 (clone RMT3-23), and PerCPCy5.5-conjugated anti-γδTCR (clone GL3, all from Biolegend). Aqua amine reactive dye (Invitrogen) was used for dead cell exclusion. Samples were analyzed on a four-laser Fortessa flow cytometer (BD Biosciences). Data analysis was performed using FlowJo software (Tree Star, Ashland, OR, USA). Boolean gating was utilized to evaluate multiparametric expression, and fluorescence minus one (FMO) controls were used to set gates. The gating strategy is illustrated in Additional file 2: Figure S2.

### Intracellular cytokine staining

PBMCs (2 × 10^5^ cells/well in triplicate) were stimulated with either uninfected RBCs (uRBCs) or iRBCs (6 × 10^5^/well) for 24 h or isopentenyl pyrophosphate (IPP, 3 μM, Sigma-Aldrich, St Louis, MO, USA) for 16 h. Brefeldin A (10 μg/ml, Sigma-Aldrich) and monensin (BD Biosciences) were added to the cells for the last 8 h of incubation. Assessment of γδ T cell cytokine production was performed by intracellular cytokine staining using allophycocyanin (APC)-conjugated anti-IFN-γ (clone B27, BD Biosciences) and PE-Cy7-conjugated anti-tumor necrosis factor alpha (TNF-α) (clone MAb11, eBioscience, San Diego, CA, USA), and cytotoxic capacity was assessed by Brilliant Violet 421-conjugated anti-CD107a (clone H4A3, Biolegend) staining in culture. A positive response was determined as the frequency of responding cells, which was twice above background and was ≥0.1% IFN-γ, TNF-α, or CD107a-positive γδ T cells of all γδ T cells or ≥0.5% positive γδ T cells of γδ T cell subsets following subtraction of background.

### Cytokine and antigen stimulation of PBMCs

PBMCs (5 × 10^5^ cells) from healthy individuals were stimulated with the following conditions: IL-6 (10 ng/ml), IFN-γ (10 ng/ml), IL-12/IL-18 (50 ng/ml each), IL-4 (10 ng/ml), IL-1β (1 ng/ml, all from Peprotech, NJ), TNF-α (10 ng/ml, Life Technologies, Carlsbad, CA, USA), iRBCs (3 iRBCs: 1 PBMC), lipopolysaccharide (LPS, 1 ug/ml, InvivoGen, San Diego, CA, USA), IPP (3 μM), or cells in medium only. After 24 h of incubation, the frequency of TIM3+ γδ T cells was assessed by flow cytometry.

### Enzyme-linked immunosorbent assay (ELISA)

Plasma was assessed for IL-12p70 and IL-18 cytokine levels using ELISA (RayBiotech, Norcross, GA, USA) according to the manufacturer’s instructions. Plasma from healthy controls were included as negative controls for IL-12 and to measure the baseline plasma IL-18 concentration of healthy individuals.

### Statistical analysis

Statistical analyses were performed using Prism 6.0 (GraphPad software) and STATA 12. Flow cytometry data were analyzed using the Student’s *t* test or Kruskal-Wallis test followed by the Dunn post-test as indicated. Correlation coefficients were determined by Spearman rank correlation. Logistic regression was used to test whether recent infection was associated with an increased proportion of TIM3+ γδ T cells and whether TIM3 expression varied the odds of experiencing a clinical malaria episode during the follow-up period. Associations between molecular force of infection (_mol_FOI) and the proportion of TIM3-expressing cells were tested using negative binomial regression and in a Cox proportional hazards model for time to first clinical episode. In order to normalize the TIM3 expression levels, the data were power transformed. Clinical incidence was defined as the frequency of occurrence per time at risk of infections associated with a fever. _mol_FOI was determined by genotyping all samples for merozoite surface protein 2 (*msp2*) using capillary electrophoresis for fragment sizing [54, 55] in addition to using PCR conditions for highly purified DNA [56]. _mol_FOI was defined as the frequency of acquisition of new malaria infections per time at risk [55]. New infections were defined as those where the detected allele had not been observed in a child at the previous two active or passive case detection visits [49]. Time at risk was adjusted for missed visits, and children were censored from the analysis after they missed three or more active case detection visits. Multivariable analyses to determine the association of TIM3 levels and relevant covariates, including IL-18 levels, were conducted using general linear models (GLMs). Backwards elimination was applied to construct the most parsimonious model.

## Results

### TIM3 expression is upregulated on γδ T cells following acute infection

**Fig. 1:**
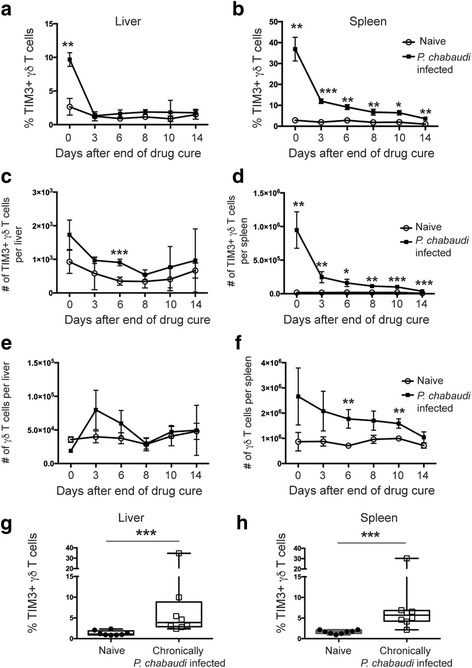
TIM3 expression is maintained on γδ T cells after drug treatment and parasite clearance. C57BL/6 mice were infected with *P. chabaudi* and then drug-treated with chloroquine and pyrimethamine. (**a**) Liver lymphocytes and (**b**) splenocytes were stained for TIM3 expression at different time points following end of drug cure to assess the percentage of TIM3+ γδ T cells, (**c**) and (**d**) number of TIM3+ γδ T cells, and (**e**) and (**f**) the total number of γδ T cells in the liver and spleen. The data represent three mice per time point and shows mean ± standard deviation (*SD*). Chronically *P. chabaudi*-infected mice (*n* = 8) were assessed on day 98 post-infection for TIM3+ γδ T cells in the (**g**) liver and (**h**) spleen. The data represent two independent experiments. Statistical analysis was performed using (**a**-**f**) paired *t* tests with Holm-Sidak method or (**g**) and (**h**) Student’s *t* test. *P < 0.05, **P < 0.01, ***P < 0.001

TIM3 expression is present on the surface of activated αβ T cells following stimulation and acts as a negative regulator [4, 39–41]. Recently TIM3 expression on conventional T cells was demonstrated to be upregulated during acute *Plasmodium* infection in both mice and humans [13, 46]. In contrast, little is known about TIM3 expression on γδ T cells and whether *Plasmodium* infection is associated with γδ T cell TIM3 expression. It is also not established whether TIM3+ γδ T cells remain detectable even in the absence of an active infection or whether chronic infection is required. Prolonged expression of negative regulators such as TIM3 may affect responses to subsequent infections. This may be especially important in settings where an individual is repeatedly infected with a pathogen, such as in malaria endemic areas. To address these questions, we examined TIM3 expression on γδ T cells following acute malaria infection. To that end, C57BL/6 mice were infected with *P. chabaudi* iRBCs, and 14 days post-infection, mice were drug-treated to resolve infection completely. Cells were isolated from the liver and spleen at different time points (0–14 days) following completion of drug treatment, and γδ T cells were subsequently assessed for TIM3 expression (Fig. 1a and b). We found that the frequency of TIM3+ γδ T cells was significantly increased in both liver (mean 9.69 ± 1.01) and spleen (mean 36.9 ± 5.66) immediately following resolution of acute infection compared to naive mice (liver: 2.66 ± 1.25 and spleen: 2.84 ± 0.45). The presence of these cells remained significantly increased in the spleen for up to 14 days (mean 3.61 ± 0.33, Fig. 1b), whereas TIM3 expression by hepatic γδ T cells returned back to background levels by day 3 post-completion of drug treatment (mean 1.77 ± 0.53, Fig. 1a). Similarly, the total number of TIM3+ γδ T cells in the spleen significantly increased from day 0 after drug treatment (mean 9.5 × 10^5^ ± 2.7 × 10^5^ cells, Fig. 1d) compared to naive mice (mean 1.8 × 10^4^ ± 6.3 × 10^3^ cells) and remained significantly increased until the last measured time point at day 14. However, the total number of all γδ T cells in the spleen at day 0 was not significantly different from that of naive mice (Fig. 1f). In contrast, the number of TIM3+ γδ T cells and the total number of all γδ T cells in the liver were not significantly increased compared to naive mice, apart from day 3 when a small increase in the number of TIM3+ γδ T cells was observed (Fig. 1c and e). To determine whether a TIM3+ γδ T cell population was persistently detectable in untreated, chronically *P. chabaudi*-infected mice, γδ T cells from these mice were assessed on day 98 p.i (Fig. 1g and h). We found that TIM3+ γδ T cells were present in both liver and spleen at a significantly higher frequency compared to naive mice (*P* = 0.0002 and *P* = 0.0006 respectively). An outlier, which had a high frequency of TIM3+ γδ T cells in both organs, was observed. Although at the time of analysis parasitemia was submicroscopic in this mouse, a recent recurrence of parasitemia is likely to have occurred. The difference in frequency of TIM3+ γδ T cells between the remaining chronically infected mice compared to naive mice remained significant when the outlier was excluded from the analysis (*P* = 0.0003 and *P* = 0.0012 respectively). This demonstrates that acute malaria infection induces γδ T cell TIM3 expression, and a population of TIM3+ γδ T cells is continually detectable both during chronic infection and following resolution of infection.

### The frequency of TIM3+ γδ T cells is increased in children living in malaria endemic areas

**Fig. 2:**
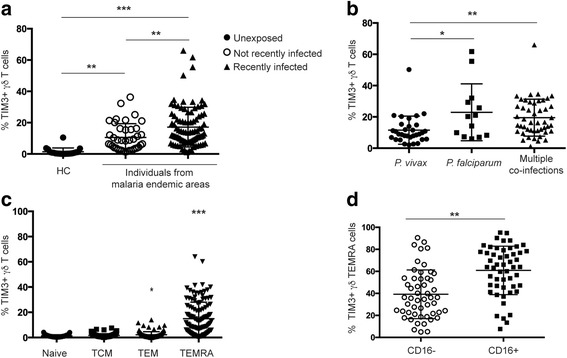
γδ T cell TIM3 expression in individuals from malaria endemic areas is increased compared to healthy controls (*HC*). PBMCs from individuals living in malaria endemic areas recently infected in the last 30 days, not recently infected, and healthy controls (HC) were surface stained for TIM3 expression. **a** Frequency of γδ T cells expressing TIM3 (recently infected, *filled triangles*, *n* = 92; not recently infected, *open circles*, *n* = 40; and healthy controls, *filled circles*, *n* = 20). **b** Percentage of γδ T cells expressing TIM3 in individuals recently infected with either *P. vivax* only (*filled circles*; *n* = 32), *P. falciparum* only (*filled squares*; *n* = 13), or co-infected with multiple species (*P. vivax, P. falciparum, P. ovale*, and *P. malariae*) (*filled triangles*; *n* = 47). **c** Frequency of TIM3+ γδ T cell subsets distinguished by expression of CD27 and CD45RA (*TCM* central memory T cell, *TEM* effector memory T cell, *TEMRA* terminally differentiated effector memory T cell). **d** Frequency of TIM3-expressing CD16+ (*open circles*) and CD16– (*squares*) TEMRA γδ T cells (mean ± SD). Statistical analysis was performed using (**a**–**c**) Kruskal-Wallis tests with Dunn’s post-test; **c** multiple comparison to naive, and (**d**) paired Student’s *t* tests.*P < 0.05, **P < 0.01, ***P < 0.001

TIM3 expression is commonly absent on γδ T cells during steady state. However, we show that TIM3 expression is induced in *Plasmodium*-infected mice. To extend these observations to human malaria, TIM3 expression was examined on γδ T cells from PBMC isolated from drug-treated children residing in malaria endemic areas in PNG (*n* = 132). We found that despite the absence of active malaria infection, all PNG children exhibited significantly higher proportions of TIM3+ γδ T cells compared to healthy controls (HC) (Fig. 2a). In addition, children who had experienced a recent infection at enrollment (28–30 days prior to PBMC collection) had a significantly increased frequency of TIM3-expressing γδ T cells compared to children who were malaria free at enrollment (*P* ≤ 0.001). In contrast, no expression of PD1 was observed on γδ T cells, but it was expressed at low frequencies on CD3+ γδTCR– cells (Additional file 3: Figure S3). This indicates that malaria exposure induces TIM3 expression, which is maintained for a considerable duration even in the absence of new infections.

To assess whether the infective *Plasmodium* species affected TIM3 expression, we compared the frequency of TIM3+ γδ T cells between children who were infected with *P. vivax* or *P. falciparum* at enrollment or had a co-infection of *P. vivax, P falciparum, P. ovale*, or *P. malariae* with a minimum of two species present. We found that TIM3+ γδ T cell was significantly increased in children with either *P. falciparum* single infections (*P* ≤ 0.05) or co-infections (*P* ≤ 0.01) compared to *P. vivax* infections alone (Fig. 2b). This suggests that infections with different species result in dissimilar TIM3 expression profiles.

γδ T cells are present in the periphery in a “pre-activated” state and can be divided into subsets based on expression of surface markers CD27 and CD45RA [57–60]. To assess which subsets of γδ T cells expressed TIM3, we distinguished between naive (CD27+ CD45RA+), central memory (TCM; CD27+ CD45RA–), effector memory (TEM; CD27– CD45RA–), and terminally differentiated effector memory (TEMRA; CD27– CD45RA+) γδ T cells (Fig. 2c, Additional file 2: Figure S2). We found that TIM3 was predominantly expressed on TEMRA γδ T cells. A subset of TEMRA cells have previously been found to express the low affinity FC receptor CD16 which defines a functionally discrete effector population, with high cytokine and cytotoxic capacity [61]. To determine the extent of TIM3 and CD16 co-expression, additional phenotypic analysis of the TEMRA subset was performed. We found that the majority of TIM3 expression was present on CD16+ TEMRA γδ T cells (Fig. 2d, A﻿dditional file 2﻿: Figure S2﻿). This demonstrates that TIM3 is primarily expressed by a memory γδ T cell population with specific function.

### γδ T cell TIM3 expression is associated with exposure to distinct parasite species and clones

We have shown that TIM3 expression differs in children infected with different *Plasmodium* species (Fig. 2b). To better understand the relationship between malaria exposure and TIM3 expression levels, the incidence of new clones acquired by each child during the follow-up period (_mol_FOI) was tested for association with TIM3. We found that expression levels on CD16+ TEMRA γδ T cells specifically were found to be significantly associated with _mol_FOI for *P. falciparum* (Table 1 and Additional file 4: Table S1, adjusted incidence rate ratio (IRR): 1.40, 95% confidence interval (CI): 1.04–1.88, *P* = 0.027), for each increment of power transformed TIM3 expression after adjusting for potential confounders: age, sex, treatment arm, infection status at enrollment, and hemoglobin level. No significant association was found with _mol_FOI for *P. vivax.* This suggests that in endemic areas, exposure to diverse parasites is a contributing factor for TIM3 upregulation in innate cells.

**Table 1:** The most parsimonius model for negative binomial regression for TIM3+ CD16+ TEMRA γδ T cell frequency and _mol_FOI

*P. falciparum* force of infection	IRR	*P*	95% CI	
PQ treatment arm	0.74	0.035	0.57	0.98
% TIM3+ CD16+ TEMRA γδ T cell (power transformed)	1.40	0.027	1.04	1.88
Hb at sampling	0.85	<0.001	0.77	0.93
Recent *P. falciparum* (*P. falciparum* infection at enrollment)	1.85	<0.001	1.39	2.46

**Fig. 3:**
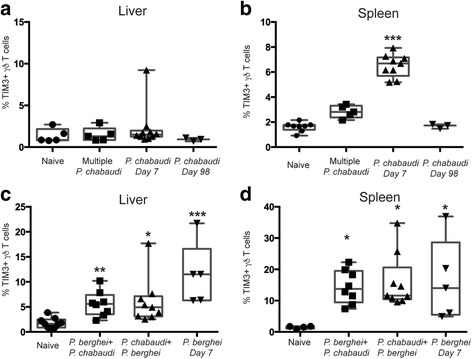
Infection and exposure to multiple malaria species are associated with upregulated TIM3 expression. C57BL/6 mice were infected with *P. chabaudi* and then drug-treated with chloroquine and pyrimethamine. (**a**) Liver lymphocytes and (**b**) splenocytes were stained for TIM3 expression on day 7 following completion of drug cure to assess the percentage of TIM3+ γδ T cells from mice which received either three sequential *P. chabaudi* infections (Multiple *P. chabaudi*, *n* = 5), single *P. chabaudi* infection (*P. chabaudi* Day 7, *n* = 9), or single *P. chabaudi* infection 98 days prior to assessment (*P. chabaudi* Day 98, *n* = 3). C57BL/6 mice were infected with either *P. chabaudi* or *P. berghei* and then drug-treated. (**c**) Liver lymphocytes and (**d**) splenocytes were assessed for TIM3+ γδ T cells on day 7 following end of drug cure from mice which received either *P. chabaudi* infection followed by *P. berghei infection* (*P. chabaudi + P. berghei*, *n* = 8), *P. berghei* infection followed by *P. chabaudi* infection (*P. berghei + P. chabaudi*, *n* = 9), or single *P. berghei* infection (*P. berghei* Day 7, *n* = 5). The data represent two independent experiments. Statistical analysis was performed using Kruskal-Wallis tests with Dunn’s post-test; comparison to naive mice. *P < 0.05, **P < 0.01, ***P < 0.001

To test the effect of consecutive infections with the same *Plasmodium* species or different species on TIM3 expression experimentally, a mouse infection model was used where C57BL/6 mice were repeatedly infected with *P. chabaudi* or *P. berghei* with drug treatment between infections. Livers and spleens were removed on day 7 after completion of the final round of drug treatment and assessed for TIM3+ γδ T cells (Additional file 1: Figure S1A). We found that mice repeatedly infected (three infections) with the same species (multiple *P. chabaudi*) did not have an increased frequency of TIM3+ γδ T cells in the spleen or in the liver (Fig. 3a and b) compared to naive mice. Control groups of mice received a single infection of *P. chabaudi* and were either drug-treated on day 14 (*P. chabaudi* day 7) or day 98 post-infection (*P. chabaudi* day 98, Additional file 1: Figure S1C) and then assessed for TIM3+ γδ T cells 7 days post-drug treatment. Consistent with Fig. 1a and b, TIM3+ γδ T cells were observed in the spleens of *P. chabaudi* day 7 mice, but not in the liver. To investigate if multiple infections with different species had an effect on TIM3 expression, we infected mice with *P. chabaudi* followed by drug treatment and then *P. berghei* infection (*P. chabaudi* + *P. berghei)* or vice versa (*P. berghei + P. chabaudi)* (Additional file 1: Figure S1B and Fig. 3c and d). In contrast to mice exposed multiple times to a single malaria species, mice infected sequentially with different species had an increased proportion of TIM3+ γδ T cells in both the spleen and liver compared to naive mice (*P* ≤ 0.05 and *P* ≤ 0.05 respectively). TIM3+ γδ T cells were also observed following a single infection of *P. berghei* at day 7 post-drug treatment completion. Collectively, these observations show that TIM3 is induced following a single infection, but not by sequential infections with the same species and clones. However, consecutive infections with different species or different clones from the same species (as indicated in Table 1) contribute to TIM3 expression in innate cells during repeated exposures.

### TIM3+ γδ T cells are dysfunctional and are associated with lower risk of acquiring clinical episodes

To investigate the relationship between expression of TIM3 and cytokine and cytotoxic capacity, the functional capacity of TIM3+ γδ T cells was determined in a subset of children where cells were available. Cytokine production and cytotoxic activity was assessed following stimulation with iRBCs or the phosphoantigen isopentenyl pyrophosphate (IPP). The predominant subsets that produce either IFN-γ or TNF-α following these stimulation conditions were TCM and TEM, collectively here referred to as memory γδ T cells, whereas cytotoxic activity was mainly observed in TEM and TEMRA subsets. The overall magnitude of the responses of all γδ T cells following background subtraction is summarized in Additional file 5: Table S2.

**Fig. 4:**
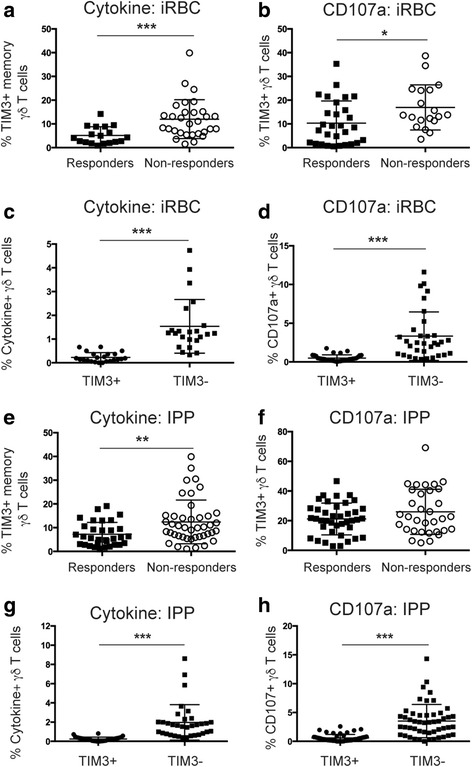
TIM3+ γδ T cells are functionally inactive following stimulation with iRBCs and IPP. PBMCs from individuals living in malaria endemic areas were stimulated with iRBCs and then surface stained for TIM3 expression. Frequency of TIM3+ γδ T cells in (**a**) and (**e**) cytokine responders and non-responders and (**b**) and (**f**) in individuals with or without cytotoxically active γδ T cells after stimulation with iRBC or IPP (responders; *filled squares* and non-responders; *open circles*). Comparison of TIM3 expression by (**c**) and (**g**) cytokine-producing γδ T cells and (**d**) and (**h**) cytotoxic γδ T cells (TIM3+; *filled circles* and TIM3–; *filled squares*) in iRBC- or IPP-responding individuals. Statistical analysis was performed using (**a**, **b**, **e**, and **f**) Mann-Whitney tests and (**c**, **d**, **g**, and **h**) Wilcoxon matched pairs signed-rank test. *P < 0.05, **P < 0.01, ***P < 0.001

Children were divided into responders and non-responders. A positive response was determined as cytokine production and cytotoxic activity following stimulation, which was twice above background and with a frequency of ≥0.1% positive γδ T cells of all γδ T cells or ≥0.5% positive γδ T cells of γδ T cell subsets following subtraction of background. Representative FACS plots of responses are presented in Additional file 6: Figure S4. We found that children in which γδ T cells did not produce cytokine (non-responders, *n* = 30) or lacked cytotoxic activity (*n* = 33) following stimulation with iRBCs had a significantly higher proportion of TIM3+ γδ T cells compared to individuals where γδ T cells responded to antigenic stimuli (Fig. 4a and b; cytokine responders *n* = 21, *P* = 0.0005 and CD107a responders *n* = 31, *P* = 0.015). Comparable results were observed with cytokine production following IPP stimulation, but not with cytolytic γδ T cells (Fig. 4e and f). Further analysis assessing TIM3 expression of all IFN-γ+, TNF-α+, and CD107a+ γδ T cells following stimulation with iRBCs (Fig. 4c and d) or IPP (Fig. 4g and h) revealed that effectively all cells that produce cytokines and exerted cytotoxic activity do not express TIM3 (Additional file 6: Figure S4). Collectively, this demonstrates that TIM3+ γδ T cells do not produce IFN-γ or TNF-α and lack cytotoxic activity in response to iRBC or phosphoantigen. Thus, the presence of these cells in the periphery of individuals may significantly affect their ability to respond to pathogens.

**Fig. 5:**
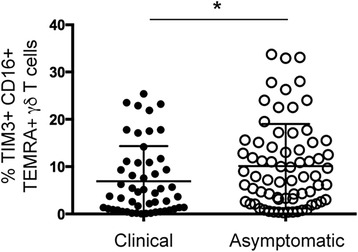
Children who experience a clinical episode during follow-up have decreased frequency of TIM3+ CD16+ TEMRA γδ T cells. Frequency of TIM3+ CD16+ TEMRA γδ T cells in children with a clinical episode (*filled circles*; *n* = 50) versus children with asymptomatic infection (*open circles*; *n* = 72) during follow-up. Statistical analysis was performed using Mann-Whitney tests. *P < 0.05

To further understand the relevance of these processes for the development of symptomatic malaria, associations between TIM3 expression on γδ T cells and the occurrence and incidence of clinical malaria during the follow-up period were explored. Significantly higher frequencies of CD16+ TIM3+ TEMRA γδ T cells were found in individuals who only experienced an asymptomatic infection (*n* = 70) compared to children who had at least one clinical episode (Fig. 5, *n* = 50, *P* = 0.022). In addition in a proportional hazards model adjusting for other covariates, the frequency of the CD16+ TIM3+ TEMRA γδ T cells was also associated with a longer time to first clinical episode (Table 2 and Additional file 7: Table S3, hazard ratio: 0.54, 95% CI: 0.30–0.95, *P* = 0.032). Together these results indicate that the presence of CD16+ TIM3+ TEMRA γδ T cells is a predictor of favorable disease outcome.

**Table 2:** The most parsimonius model for proportional hazard model for TIM3+ CD16+ TEMRA γδ T cell frequency and time to first clinical malaria episode

	Hazard ratio	*P* > z	95% CI	
TIM3+ CD16+ TEMRA γδ T cells	0.54	0.032	0.30	0.95

### TIM3 expression by γδ T cells is induced by pro-inflammatory cytokines and phosphoantigens

**Fig. 6:**
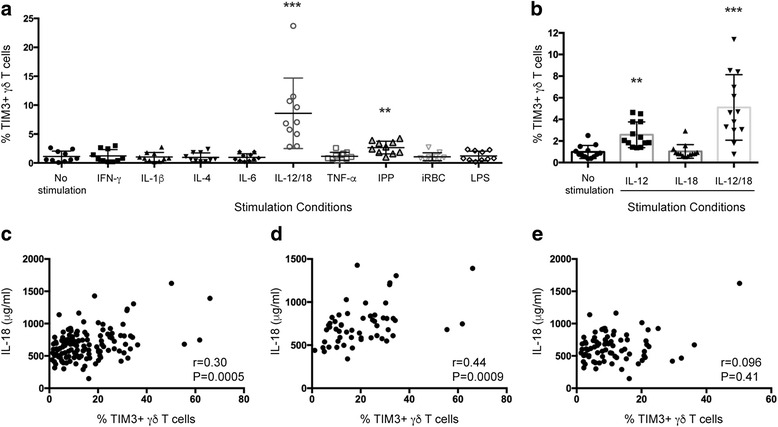
TIM3 is upregulated by IL-12/18 and IPP. PBMCs from healthy individuals (*n* = 10) were stimulated with (**a**) iRBCs (3:1), LPS, IPP, or cytokines (IL-6, IFN-γ, TNF-α, IL-12/IL-18, IL-4, IL-1β) or (**b**) IL-12 and IL-18 for 24 h and then surface stained for TIM3 expression. The frequency of γδ T cells expressing TIM3 was compared to that of unstimulated cells. Correlation of IL-18 plasma levels and TIM3 expression in (**c**) all children (*n* = 132), (**d**) children with *P. falciparum* infection at enrollment (*n* = 55), and (**e**) children with no *P. falciparum* infection at enrollment (*n* = 77). Statistical analysis was performed using Kruskal-Wallis tests with Dunn’s post-test (**a** and **b**) and Spearman rank correlation (**c** and **d**). **P < 0.01, ***P < 0.001

During *Plasmodium* infection a range of cytokines are induced which are associated with both protective responses as well as clinical outcomes and are also important for either a Th1 or Th2 response [62]. To determine if parasite-specific stimuli or specific cytokines related to malaria infection induce TIM3 expression on γδ T cells, we stimulated PBMCs from healthy individuals in vitro with cytokines (IL-6, IL-1β, TNF-α, IL-12/IL-18, IL-4, and IFN-γ), IPP, iRBCs, or LPS for 24 h. TIM3 was detected on the surface of γδ T cells only in the presence of IPP and IL-12/18 (Fig. 6a). Furthermore, IL-18 alone was unable to upregulate TIM3, but it augmented IL-12-induced TIM3 expression (Fig. 6b). As only IL-12/IL-18 induced TIM3 expression on γδ T cells, the plasma levels of these TIM3-inducing cytokines were investigated in the PNG children. Interestingly, plasma IL-18 levels were correlated with TIM3 expression (Fig. 6c; *r* = 0.30, *P* = 0.0005). This correlation remained significant in recently *P. falciparum*-infected children (Fig. 6d; *r* = 0.44, *P* = 0.0009) and in multivariable analyses (Table 3 and Additional file 8: Table S4, coefficient 0.03, 95% CI: 0.009–0.04, *P* = 0.002), whereas this association was not observed in non-*P. falciparum*-infected children (Fig. 6e; *r* = 0.096, *P* = 0.41), suggesting that IL-18 levels are driven by *P. falciparum* infection. In contrast, plasma IL-12 levels were undetectable in the majority of children at this time point.

**Table 3:** The most parsimonius model for general linear model of TIM3+ γδ T cell frequency with IL-18 levels in children with recent *P. falciparum* infection

	Coefficient	*P*	95% CI	
IL-18 (μg/ml)	0.03	0.002	0.009	0.04

## Discussion

The significant contribution of γδ T cells to the overall immunity during infection and cancer is increasingly appreciated. Although recent efforts to utilize γδ T cells as immunotherapy effector cells have produced promising results [63], it is evident that a more comprehensive understanding of the biology related to immunoregulation of these cells is required to overcome the demonstrated induction of anergy and exhaustion upon repeat exposure to antigens [64, 65]. Our data demonstrated that in an infectious disease setting with continuous exposure to malaria, TIM3 expression becomes upregulated on γδ T cells, and this process is controlled by environmental cues provided by the host immune response. IFN-γ, TNF-α, and cytotoxic activity were found to be absent in TIM3+ γδ T cells upon re-stimulation with malaria antigens, and increased frequencies of these cells were associated with reduced risk of clinical malaria.

Although *P. vivax* is considered less virulent than *P. falciparum*, *P. vivax* is still a major cause of morbidity in endemic areas. Nevertheless, substantial differences in immunity to the two species have been noted, in particular in regard to naturally acquired immunity, where immunity to *P. falciparum* is slower to develop than immunity to *P. vivax* [66]*.* Few studies have compared innate responses and cytokine profiles between these species, though dissimilarities are plausible considering the distinct naturally acquired immunity patterns. We observed that recent infection with either *P. vivax* or *P. falciparum* affected the frequency of TIM3+ γδ T cells. However, the extent of TIM3 upregulation was different depending on the infective species. CD4+ T cells upregulate TIM3 following extended stimulation with IL-12 [67]. Similarly, we also found that IL-12/IL-18 can promote γδ T cell upregulation of TIM3 in vitro where IL-18 plays an auxiliary role. While both *Plasmodium* species are associated with increases in IL-12 production during acute infection, *P. falciparum* infection has been demonstrated to result in higher plasma IL-12 levels during convalescence [68, 69]. In the current cohort IL-12 was undetectable in the plasma, which is likely due to the absence of active infection for 28–30 days preceding the time point for PBMC collection. However, IL-18 plasma levels were readily detectable and were associated with TIM3 expression in *P. falciparum*-infected children at enrollment. Thus, dissimilar cytokine profiles and concentration levels at the time of infection may contribute to the difference in TIM3+ γδ T cell frequencies observed between children recently infected with *P. vivax* and *P. falciparum*. Notably, IL-12/IL-18 can also induce IFN-γ production by γδ T cells. However, the effect of IL-12/IL-18 on TIM3 induction by γδ T cells may be dose-dependent, as was previously shown for TIM3 expression on CD4+ T cells [67]. Thus, early production of IL-12/IL-18 may promote IFN-γ production, whereas accumulation of these cytokines in the plasma may induce γδ T cells to upregulate TIM3. Furthermore, while no association was observed between IL-18 plasma levels in children who were not infected with *P. falciparum* and TIM3 expression, these children still have an increased population of TIM3+ γδ T cells. It is possible that phosphoantigen contributes to the observed TIM3 expression in these children, as IPP was also observed to induce TIM3 expression, albeit at lower frequency. However this remains to be determined.

Experimental mice infected with different *Plasmodium* species resulted in significant TIM3 upregulation, and TIM3+ γδ T cells remained detectable after resolution of infection. Comparable findings were observed in the PNG children where TIM3 expression was associated with _mol_FOI, thus suggesting that exposure to distinct parasites is important for induction of TIM3 during repeated exposure. Cytokine responses to malaria have been reported to be influenced by the immune status of the host [70, 71]. It is possible that the differences in cytokine profiles resulting from re-infection with the same parasite among immune hosts versus infection with a new clone or species may affect TIM3 expression in the innate cell compartment.

Continuous exposure to malaria is correlated with immunity to symptomatic disease and is likely to involve both antiparasitic mechanisms and regulation of cytokines implicated in immunopathogenesis [72]. γδ T cells play a protective role as a major IFN-γ producer during malaria infection [73] but are also a major source of cytokines and chemokines associated with disease [29]. Typically, Vγ9+ Vδ2+ T cells are considered to be the malaria-antigen responsive cells and also represent the majority of γδ T cells in peripheral blood [31, 74]. However, the functional roles for γδ T cells in general are continuously expanding, indicating that the contribution of other subsets of γδ T cells to both malaria immunity and immunopathogenesis may not be completely appreciated. Therefore, in contrast with the previous studies, we investigated TIM3 expression on the total γδ T cell population without distinguishing subsets based on TCR restriction. In the current study, we found that the TIM3+ γδ T cells from malaria-exposed individuals were effectively unresponsive to stimulation in vitro. The cells produced minimal IFN-γ and TNF-α and demonstrated low cytotoxic activity in response to iRBCs or phosphoantigen, thus indicating that these cells were functionally impaired. Interestingly, we observed that TIM3 was predominately expressed by CD16+ TEMRA γδ T cells. Our study is aligned with recent findings suggesting that dysfunctional CD16+ γδ T cells emerge in response to malaria exposure [75]. While CD16 upregulation is most likely a consequence of prior TCR activation [76], expression of CD16 alone does not explain why these cells are impaired. Here we identified TIM3 as a potential receptor responsible for the γδ T cell impairment associated with malaria infection.

It is worth noting that the CD16+ TEMRA γδ T cell subset is reported to be unresponsive to phosphoantigen stimulation [61]. Instead CD16 allows γδ T cells to recognize opsonized targets. Farrington et al. (2016) proposed that accumulation of CD16+ cells represents a population which is preferentially stimulated through this receptor independently of the TCR [75]. However, signaling through CD16 results in both TNF-α and IFN-γ production [61, 76]. Given that TNF-α is a pyrogenic cytokine that significantly contributes towards malaria febrile disease [77, 78], it indicates that inhibition of CD16+ TEMRA γδ T cells may be necessary to limit immunopathogenesis in the host. Notably, this study identifies specifically that the TIM3+ CD16+ TEMRA γδ T cell population was associated with reduced clinical incidence risk, which supports the concept that regulation of highly specialized subsets is important for reducing clinical malaria symptoms. Thus, increased TIM3 expression on this population was associated with less risk of febrile malaria and was associated with asymptomatic infections. While TIM3 may not directly inhibit CD16 signaling, a functional linkage between CD16 and TCR signaling has been reported [79]. In αβ T cells, TIM3 inhibition of TCR signaling is regulated by Bat3 interaction potentially through binding of catalytically active Lck [80]. Thus, TIM3 inhibition of the TCR may also affect the CD16-dependent response in this γδ T cell population, although this remains to be determined.

While we detected TIM3+ γδ T cells in the mouse model, in vivo assessment of their relative contribution to disease outcome using either a TIM3 knock-out mouse model or TIM3 depletion or blocking is hampered by the fact that TIM3 is known to be expressed by several different cell populations. These approaches would result in the inability to specifically assign effects on disease outcome to TIM3+ γδ T cells. Thus, a conditional knock-out mouse model would be required to address this. Furthermore, adoptive transfer of TIM3+ γδ T cells is potentially confounded by the presence of bound antibody, which may interfere with endogenous ligand interaction. Consequently, experimentally defining the role of TIM3+ γδ T cells for malaria disease outcome in vivo still remains unresolved.

## Conclusions

In conclusion, this study demonstrates the effect of continuous malaria exposure in shaping the innate immune response. The findings further emphasize the importance of regulating immune cells as part of clinical immunity to malaria. However, they also demonstrate that the inhibitory receptor TIM3 is an important regulatory component in γδ T cell biology and may have implications for disease management of malaria as well as γδ T cell immunotherapy in general.

## Additional files

Additional file 1: **Figure S1.** Schematic of single and multiple infections with *P. berghei* and/or *P. chabaudi.* C57BL/6 mice were infected with (A) *P. chabaudi* three consecutive times with drug treatment between infections, (B) *P berghei* only, *P. chabaudi* followed by drug treatment and then *P. berghei* infection or vice versa, or (C) *P. chabaudi* to establish chronic infections followed by drug treatment. (TIF 1009 kb)

Additional file 2: **Figure S2.** Gating strategy used to determine γδ T cell memory populations and TIM3 expression by γδ T cells in both PBMC samples and mice. (TIF 1730 kb)

Additional file 3: **Figure S3.** PD1 expression by γδ T cells is absent. PBMCs from individuals living in malaria endemic areas and healthy controls (*HC*) were surface stained for TIM3 and PD1 expression. (A) Frequency of γδ T cells expressing TIM3 or PD1 in individuals living in malaria endemic areas. (B) Frequency of CD3+ γδTCR– cells expressing PD1 in HC and individuals living in malaria endemic areas. Statistical analysis was performed using Paired *t* tests (A) or Mann-Whitney tests (B). ***P < 0.001. (TIF 1245 kb)

Additional file 4: **Table S1.** Negative binomial regression for TIM3+ CD16+ TEMRA γδ T cell frequency and _mol_FOI. (DOC 35 kb)

Additional file 5. **Table S2.** Frequency of γδ T cell responses following background subtraction. (DOC 30 kb)

Additional file 6: **Figure S4.** TIM3+ γδ T cells are functionally impaired following stimulation with iRBCs and IPP. PBMCs from individuals living in malaria endemic areas were stimulated with either iRBCs or IPP and then surface stained for TIM3 expression. FACS plots representing frequency of TIM3 expression on IFN-γ, TNF-α, and CD107a producing γδ T cells following stimulation with iRBCs and uRBCs (*top panel*) or IPP and no stimulation (*bottom panel*). (TIF 1308 kb)

Additional file 7: **Table S3.** Proportional hazard model for TIM3+ CD16+ TEMRA γδ T cell frequency and time to first clinical malaria episode. (DOC 35 kb)

Additional file 8: **Table S4.** General linear model of TIM3+ γδ T cell frequency with IL-18 levels in children with recent *P. falciparum* infection. (DOC 33 kb)

## Abbreviations

CQ: Chloroquine
DOT: Directly observed treatment
HC: Healthy control
IPP: Isopentenyl pyrophosphate
iRBC: Infected red blood cell
_mol_FOI: Molecular force of infection
PNG: Papua New Guinea
PQ: Primaquine
TIM3: T-cell immunoglobulin domain and mucin domain 3
uRBC: Uninfected red blood cell
