# Supplementary figures and images for: Synergistic effect of IL-12 and IL-18 induces TIM3 regulation of γδ T cell function and decreases the risk of clinical malaria in children living in Papua New Guinea
Source: BMC Med. 2017 Jun 15;15:114. doi: 10.1186/s12916-017-0883-8 (PMC5471992; doi:10.1186/s12916-017-0883-8)

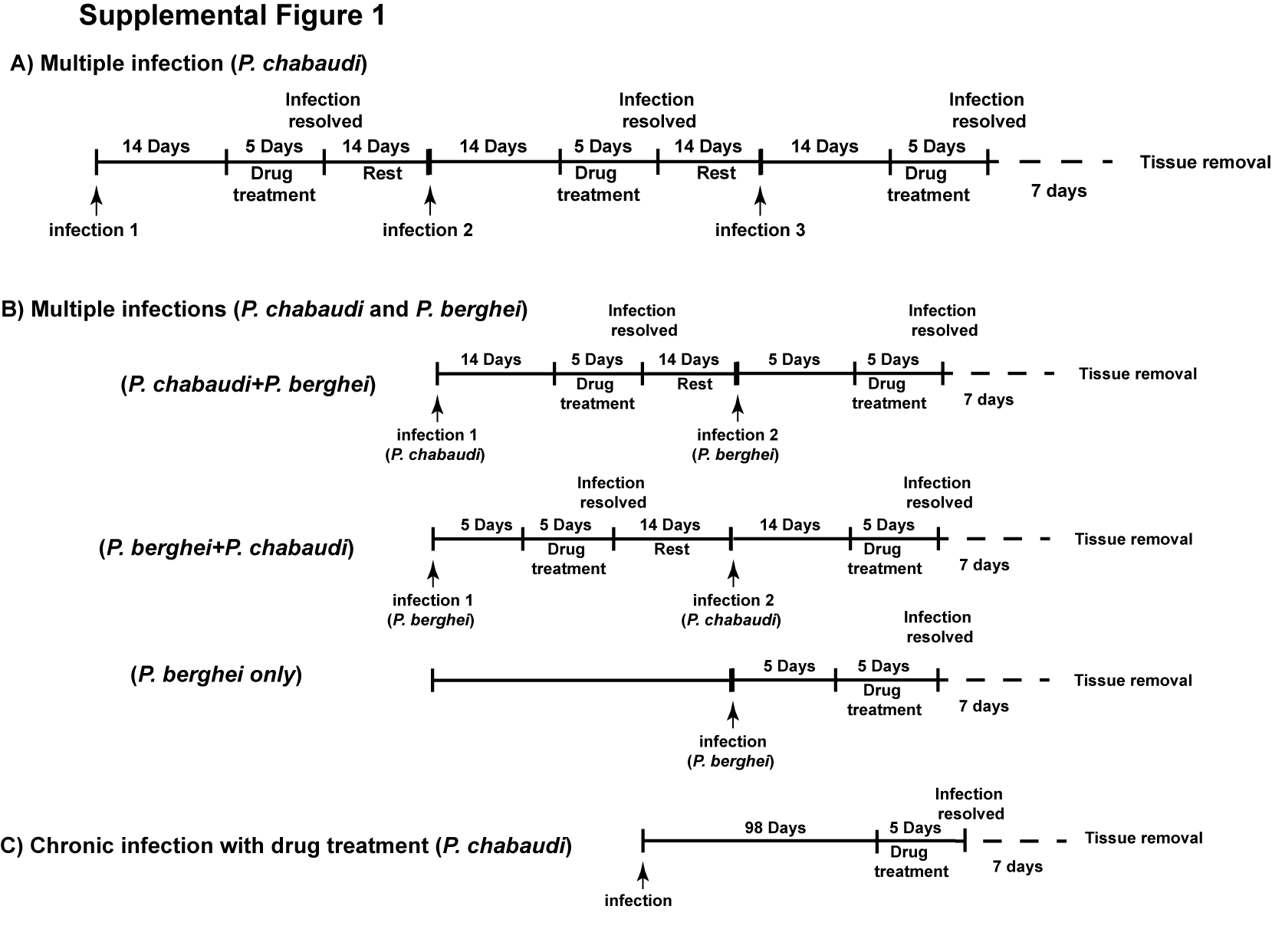

Supplement: Supplementary file 1 — Figure S1. Schematic of single and multiple infections with P. berghei and/or P. chabaudi. C57BL/6 mice were infected with (A) P. chabaudi three consecutive times with drug treatment between infections, (B) P berghei only, P. chabaudi followed by drug treatment and then P. berghei infection or vice versa, or (C) P. chabaudi to establish chronic infections followed by drug treatment. (TIF 1009 kb) [file 12916_2017_883_MOESM1_ESM.tif]

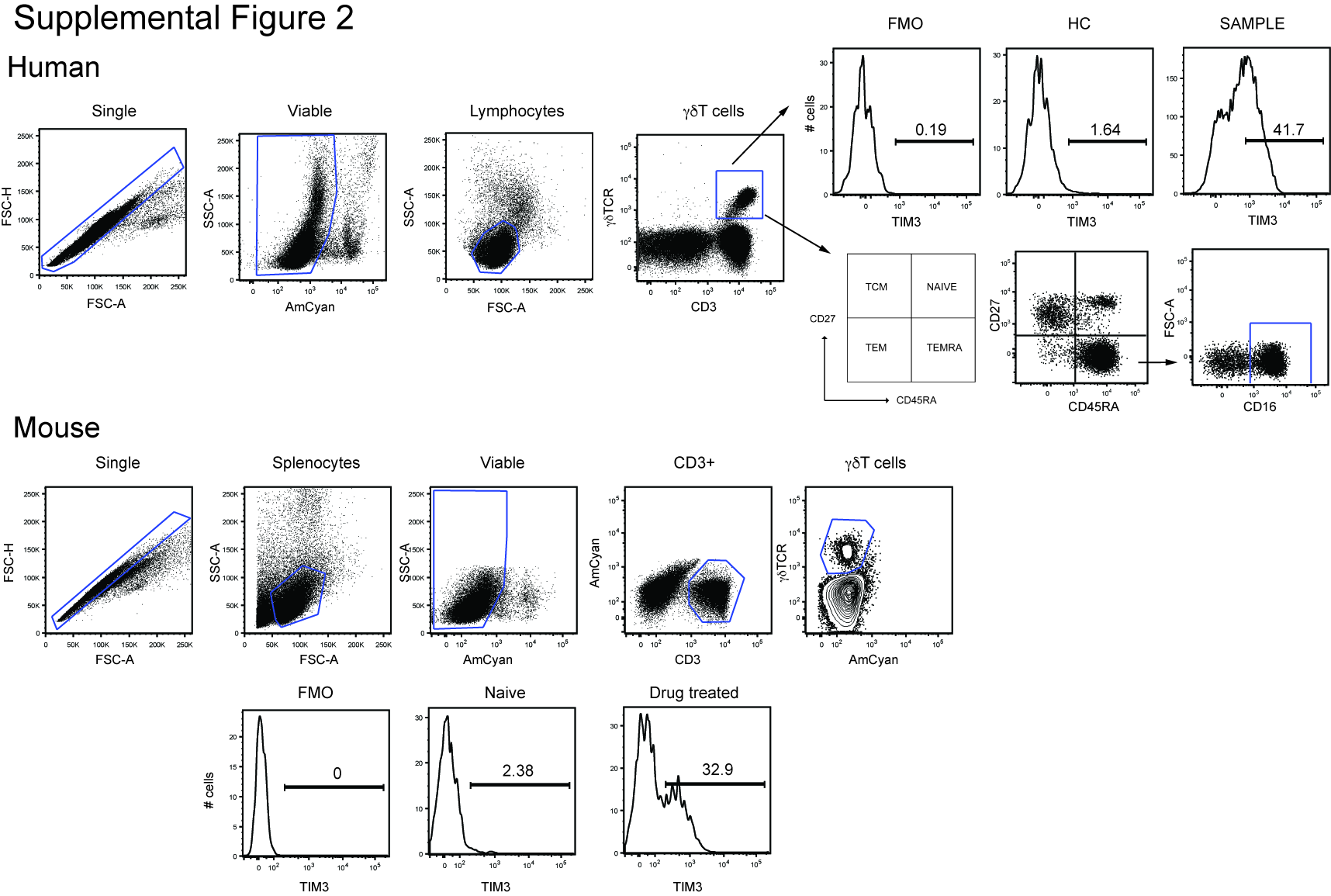

Supplement: Supplementary file 2 — Figure S2. Gating strategy used to determine γδ T cell memory populations and TIM3 expression by γδ T cells in both PBMC samples and mice. (TIF 1730 kb) [file 12916_2017_883_MOESM2_ESM.tif]

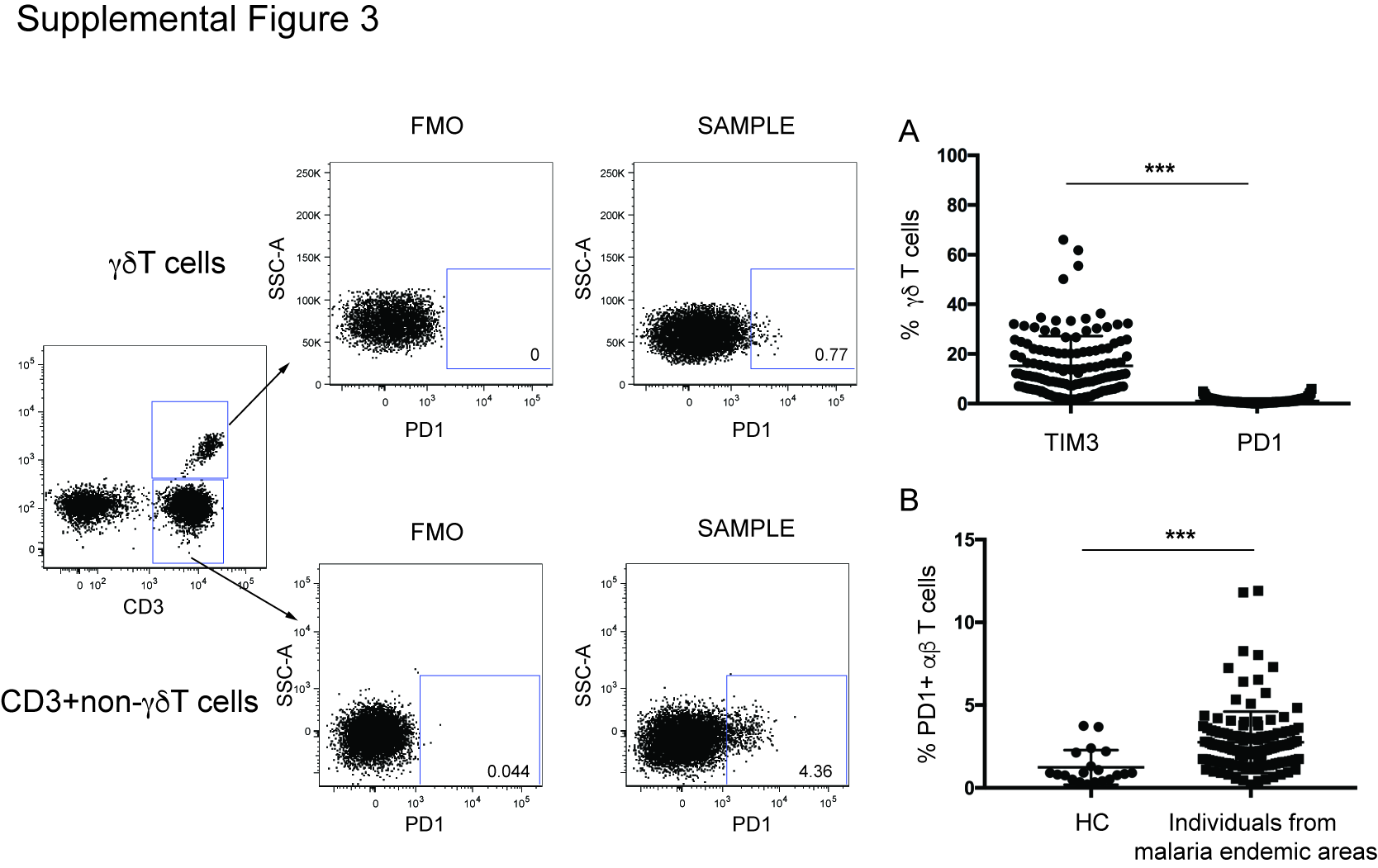

Supplement: Supplementary file 3 — Figure S3. PD1 expression by γδ T cells is absent. PBMCs from individuals living in malaria endemic areas and healthy controls (HC) were surface stained for TIM3 and PD1 expression. (A) Frequency of γδ T cells expressing TIM3 or PD1 in individuals living in malaria endemic areas. (B) Frequency of CD3+ γδTCR– cells expressing PD1 in HC and individuals living in malaria endemic areas. Statistical analysis was performed using Paired t tests (A) or Mann-Whitney tests (B). ***P < 0.001. (TIF 1245 kb) [file 12916_2017_883_MOESM3_ESM.tif]

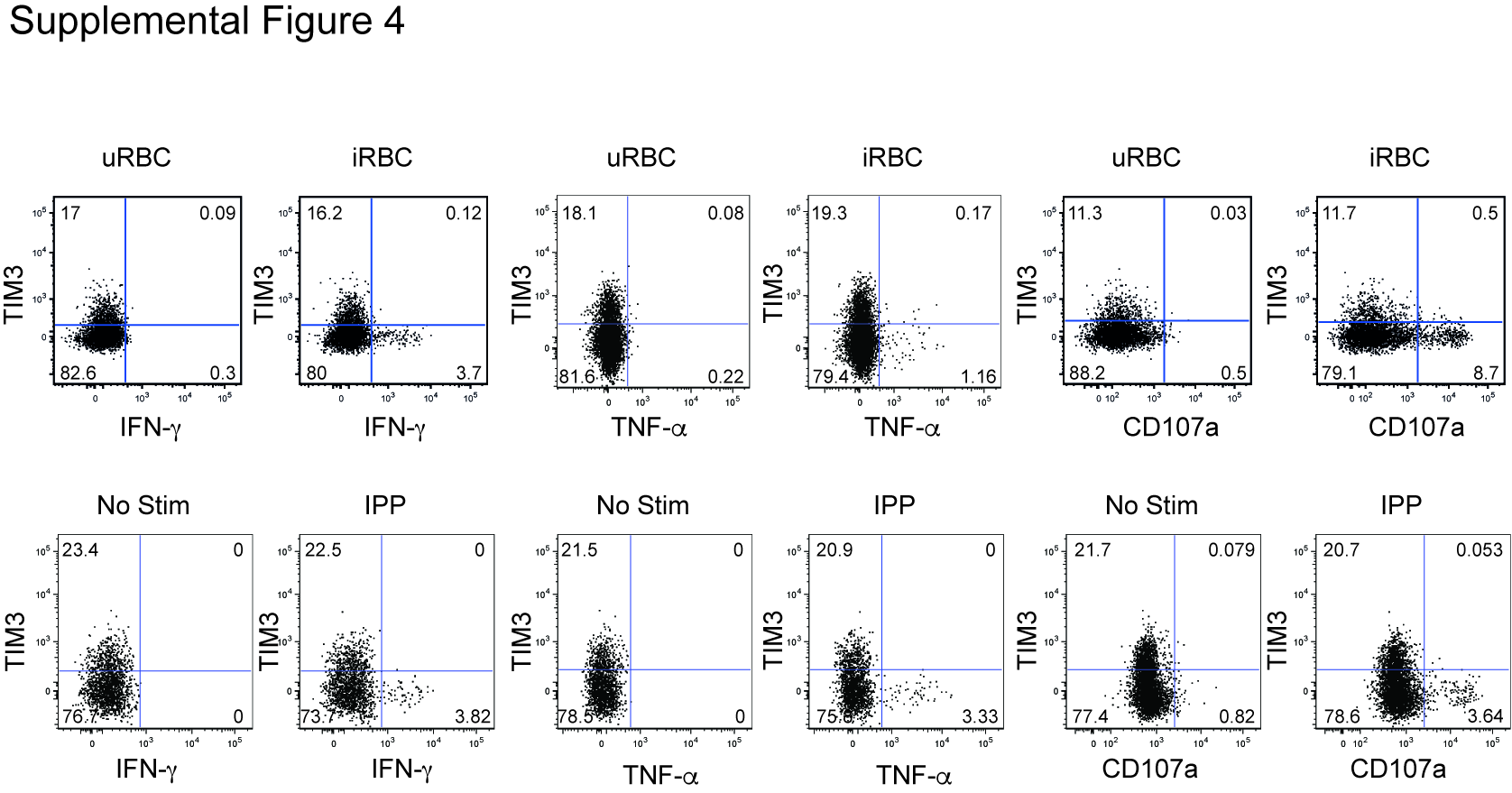

Supplement: Supplementary file 6 — Figure S4. TIM3+ γδ T cells are functionally impaired following stimulation with iRBCs and IPP. PBMCs from individuals living in malaria endemic areas were stimulated with either iRBCs or IPP and then surface stained for TIM3 expression. FACS plots representing frequency of TIM3 expression on IFN-γ, TNF-α, and CD107a producing γδ T cells following stimulation with iRBCs and uRBCs (top panel) or IPP and no stimulation (bottom panel). (TIF 1308 kb) [file 12916_2017_883_MOESM6_ESM.tif]
