# Supplementary material for: Synergistic effect of IL-12 and IL-18 induces TIM3 regulation of γδ T cell function and decreases the risk of clinical malaria in children living in Papua New Guinea
Source: BMC Med. 2017 Jun 15;15:114. doi: 10.1186/s12916-017-0883-8 (PMC5471992; doi:10.1186/s12916-017-0883-8)
Supplement: Supplementary file 4 — Table S1. Negative binomial regression for TIM3+ CD16+ TEMRA γδ T cell frequency and molFOI. (DOC 35 kb) [file 12916_2017_883_MOESM4_ESM.doc]

Table S1. Negative binomial regression for TIM3+ CD16+ TEMRA γδ T cell frequency and _mol_FOI

| ***P. falciparum* force of infection** | IRR | P | 95% CI |  |
| --- | --- | --- | --- | --- |
| PQ treatment arm | 0.74 | 0.026 | 0.57 | 0.96 |
| % TIM3+ CD16+ TEMRA γδ T cell (power transformed) | 1.43 | 0.012 | 1.08 | 1.90 |
| Hb at sampling | 0.84 | < 0.001 | 0.77 | 0.92 |
| Sex (female) | 0.89 | 0.43 | 0.67 | 1.18 |
| Age | 0.99 | 0.80 | 0.90 | 1.09 |
| recent *P. vivax*  (*P. vivax* infection at enrollment) | 0.90 | 0.43 | 0.68 | 1.18 |
| recent *P. falciparum*  (*P. falciparum* infection at enrollment) | 1.88 | <0.001 | 1.43 | 2.47 |
