# Supplementary material for: Synergistic effect of IL-12 and IL-18 induces TIM3 regulation of γδ T cell function and decreases the risk of clinical malaria in children living in Papua New Guinea
Source: BMC Med. 2017 Jun 15;15:114. doi: 10.1186/s12916-017-0883-8 (PMC5471992; doi:10.1186/s12916-017-0883-8)
Supplement: Supplementary file 5 — Table S2. Frequency of γδ T cell responses following background subtraction. (DOC 30 kb) [file 12916_2017_883_MOESM5_ESM.doc]

Table S2 Frequency of γδ T cell responses following background subtraction

| Stimulation | IFNγ  (Mean ± SD) | TNFα  (Mean ± SD) | CD107a  (Mean ± SD) |
| --- | --- | --- | --- |
| iRBC | 1.06 ± 1.09 | 0.62 ± 0.31 | 3.15 ± 3.18 |
| IPP | 1.97 ± 1.92 | 1.37 ± 0.92 | 3.35 ± 2.77 |
