# Supplementary material for: Synergistic effect of IL-12 and IL-18 induces TIM3 regulation of γδ T cell function and decreases the risk of clinical malaria in children living in Papua New Guinea
Source: BMC Med. 2017 Jun 15;15:114. doi: 10.1186/s12916-017-0883-8 (PMC5471992; doi:10.1186/s12916-017-0883-8)
Supplement: Supplementary file 7 — Table S3. Proportional hazard model for TIM3+ CD16+ TEMRA γδ T cell frequency and time to first clinical malaria episode. (DOC 35 kb) [file 12916_2017_883_MOESM7_ESM.doc]

Table S3. Proportional Hazard model for TIM3+ CD16+ TEMRA γδ T cell frequency and time to first clinical malaria episode

|  | Hazard Ratio | P>z | 95% CI |  |
| --- | --- | --- | --- | --- |
| % TIM3+ CD16+ TEMRA γδ T cells | 0.36 | 0.005 | 0.17 | 0.73 |
| Primaquine | 1.76 | 0.079 | 0.94 | 3.31 |
| Age | 0.89 | 0.256 | 0.72 | 1.09 |
| Amahu | 0.97 | 0.97 | 0.18 | 5.26 |
| Balanga | 2.82 | 0.15 | 0.68 | 11.72 |
| Balif | 2.95 | 0.10 | 0.81 | 10.79 |
| Bolumita | 2.95 | 0.097 | 0.82 | 10.61 |
| Recent *P. falciparum*  (*P. falciparum* infection at enrollment) | 1.11 | 0.81 | 0.48 | 2.57 |
| Recent *P. vivax*  (*P. vivax* infection at enrollment) | 1.38 | 0.36 | 0.69 | 2.73 |
