# Supplementary material for: Synergistic effect of IL-12 and IL-18 induces TIM3 regulation of γδ T cell function and decreases the risk of clinical malaria in children living in Papua New Guinea
Source: BMC Med. 2017 Jun 15;15:114. doi: 10.1186/s12916-017-0883-8 (PMC5471992; doi:10.1186/s12916-017-0883-8)
Supplement: Supplementary file 8 — Table S4. General linear model of TIM3+ γδ T cell frequency with IL-18 levels in children with recent P. falciparum infection. (DOC 33 kb) [file 12916_2017_883_MOESM8_ESM.doc]

**Table S4.** General linear model of TIM3+ γδ T cell frequency with IL-18 levels in children with recent *P. falciparum* infection.

|  | Coefficient | P | 95% CI |  |
| --- | --- | --- | --- | --- |
| _mol_FOI | -0.07 | 0.85 | -0.73 | 0.60 |
| IL-18 (μg/ml) | 0.03 | 0.001 | 0.01 | 0.04 |
| Age | 2.00 | 0.070 | -0.16 | 4.16 |
| recent *P. vivax*  (*P. vivax* infection at enrollment) | 1.00 | 0.825 | -7.85 | 9.85 |
| *P. falciparum* parasite density | 2.46 | 0.230 | -1.55 | 6.47 |
